# HOW HEARING DIFFICULTIES AND SOCIAL ACTIVITIES SHAPE TRAJECTORIES OF COGNITIVE FUNCTION AMONG OLDER MEXICANS

**DOI:** 10.1093/geroni/igae098.2530

**Published:** 2024-12-31

**Authors:** Chengming Han, Rebeca Wong

**Affiliations:** The University of Texas Medical Branch, Galveston, Texas, United States; The University of Texas Health Sciences Center in San Antonio, San Antonio, Texas, United States

## Abstract

Objective. This paper aims to explore the role of social activities in the pathway between hearing difficulties and cognitive function among Mexicans □ 50 years over a longitudinal period. Method. Data were drawn from the Mexican Health and Aging Study (MHAS) 2015 to 2021. MHAS is a national longitudinal study of adults □ 50 years in Mexico, with baseline in 2001, and six waves completed by 2021. The analytical sample includes 8,049 Mexicans at wave 4 (2015). Cognition was operationalized as verbal memory and visual scanning scores. Multilevel ordinary least squares regression models were used to estimate the mediating effects of social activities on the association between hearing difficulties and cognition over 2015 to 2021. Results. There are 2766 (34.36%) respondents who reported having a poor or fair hearing in 2015, among whom only 9% used hearing aids. Poor or fair hearing was associated with reduced social activities, which in turn related to decreased visual scanning scores in 2021. Poor/fair hearing showed both indirect and direct effects on visual scanning scores. Reporting more social activities was associated with better cognition independently. Using a hearing aid was associated with lower verbal memory scores. Conclusion. There are few studies on sensory limitations and cognitive function that include populations in middle-income countries like Mexico. Although the results displayed on the similar mechanism on hearing difficulties and cognition in MHAS, the lower percentage of using a hearing aid suggested that Mexico has experienced rapid population aging with inadequate institutional social support for health care.

